# Uncovering ART adherence inconsistencies: An assessment of sustained adherence among adolescents in South Africa

**DOI:** 10.1002/jia2.25832

**Published:** 2021-10-28

**Authors:** Siyanai Zhou, Lucie Cluver, Yulia Shenderovich, Elona Toska

**Affiliations:** ^1^ Centre for Social Science Research University of Cape Town Cape Town South Africa; ^2^ Department of Sociology University of Cape Town Cape Town South Africa; ^3^ Department of Social Policy and Intervention University of Oxford Oxford United Kingdom; ^4^ Department of Child and Adolescent Psychiatry University of Cape Town Cape Town South Africa; ^5^ School of Public Health and Family Medicine University of Cape Town Cape Town South Africa; ^6^ Wolfson Centre for Young People's Mental Health Cardiff University Cardiff UK; ^7^ Centre for the Development and Evaluation of Complex Interventions for Public Health Improvement (DECIPHer) School of Social Sciences Cardiff University Cardiff UK

**Keywords:** ARV, adherence, adolescents, cohort studies, viral suppression

## Abstract

**Introduction:**

Antiretroviral treatment (ART) adherence rates are lower among adolescents living with HIV (ALHIV) than among adults and children, but more evidence is needed on long‐term sustained ART adherence among ALHIV. This study assesses rates of sustained ART adherence in a cohort of adolescents in South Africa.

**Methods:**

A prospective cohort of adolescents (10‐19 years) living with HIV (baseline sample N = 1 046, 55% female, mean age 13.6) in the Eastern Cape Province in South Africa were interviewed at baseline (2014‐15) and followed‐up twice (2015‐16, 2017–18). All adolescents ever initiated on treatment in 52 government health facilities were traced (with 90% uptake, 94% retention at Wave 2, and 97% retention at Wave 3, 3.4% mortality) and their clinic records were extracted where available. We investigate sustained ART adherence among adolescents interviewed at all three waves of data collection (N = 933). To quantify adherence at each study wave, we used self‐reported past‐week adherence (including weekdays and weekends). Self‐reported adherence was validated using HIV‐1 RNA viral load (>50 copies/mL cut‐off) reported in clinic records, in a random‐intercept logistic regression.

**Results and discussion:**

At baseline, approximately 66% (N = 615) of adolescents reported past‐week ART adherence, and of these 45.3% reported adherence at both baseline and follow‐up. Only 37.1% of the sample reported sustained past‐week ART adherence over the three waves of the study. Most adolescents (N = 587, 62.9%) report inconsistent adherence across time (including 6.4% disengaged from care). Older (*P* = 0.007) and adolescents with horizontally acquired HIV (*P* = 0.002) were more likely to report inconsistent adherence across time. Controlling for socio‐demographic characteristics, past‐week adherence was associated with non‐detectable viral load (aOR 1.72, 95%CI 1.14‐2.59, *P* = 0.009). Overall, of the adolescents with viral load measurements at study Wave 1 and Wave 2, 50.6% maintained undetectable viral load for the preceding year.

**Conclusions:**

Adolescents living with HIV reported very low rates of sustained ART adherence. Adherence reported at a single time may mask high rates of variability in adherence over time. These findings highlight the urgent need for enhanced and effective interventions to assist ALHIV with ART adherence through the challenging years of adolescence.

## 1 INTRODUCTION

In 2019, an estimated 1.7 million adolescents aged 10–19 years were living with HIV (ALHIV) globally, with the majority (80%) living in sub‐Saharan Africa (SSA) [1, 2]. South Africa has the highest number of ALHIV, with an estimated 360 000 ALHIV in 2019 [2]. As more children born with HIV survive into adulthood and infections among 15–24 year‐olds continue to rise [3, 4], adolescents make up a growing portion of the global HIV burden. HIV‐related deaths among adolescents have tripled over the last two decades [5]. Adolescents continue to experience disproportionately high rates of poor antiretroviral (ART) treatment outcomes, presenting a significant challenge to global attempts to meet the UNAIDS 90‐90‐90 targets [6, 7, 8]. For example, 2016 UNAIDS estimates showed that HIV is the second leading cause of death among adolescents [9], while retention in care among adolescents in low and middle‐income countries has been reported to decrease from 88% at 12 months and 67% at 36 months [10]. Moreover, a recent systematic review by Ferrand and colleagues found that the proportion of adolescents with virological suppression at 12 months ranged from 27% to 89% [7, 11].

Several studies have reported lower adherence rates among adolescents than in children and adults [3, 12‐14], which increases their risk of viral rebound and drug resistance, leading to treatment failure [14, 15]. Results from the limited number of adherence studies conducted among adolescents in SSA have often found suboptimal adherence to ART and poor virologic outcomes [14]. This is largely due to several barriers to adherence among adolescents, as underscored in several reviews [16, 17]. A small cohort study (N = 154) of adolescents with horizontally acquired HIV (11‐19 years) in nine Southern African countries found that few (20.7%) adolescents achieved ≥95% adherence at 6 months and sustained adherence over time decreased to 14.3% at 12 months and 6.6% at 24 months [14]. Another cohort study (N = 250) of ALHIV aged 12–18 years in Asia, assessing long‐term adherence over 3 years, found that the proportion of ALHIV reporting adherence ≥95% decreased from 69% at baseline to 60% at 36 months [18]. Recent evidence from a cohort study (N = 179) in the United States among adolescents aged 13–24 years followed up over 5 years, highlights a decrease in the proportion of adolescents reporting past‐week adherence from 65% at the 12 months to 58% at 48 months [19]. These rates vary across studies and evidence on sustained adherence among adolescents in South Africa remains scarce [20]. Moreover, most evidence available globally on ALHIV adherence is based on fairly small samples.

While the viral load is an objective measure of adherence and one of the most accurate ways of assessment, it is not routinely available in many resource‐limited settings [21]. Self‐reported measures of adherence remain common in both social surveys and clinical settings and may assist to identify adolescents at risk of virologic failure and poor health [15]. Given the scale of the epidemic in the region, particularly among adolescents and young people in South Africa, it is crucial to understand the rates of sustained ART using longitudinal designs and rigorous measures of adherence [16, 22]. Therefore, this study utilized the self‐reported measures of adherence to describe rates of sustained ART adherence in ALHIV and assess consistency in adherence over the three waves of data collection within a large cohort of adolescents.

## 2 METHODS

This report used data from the Mzantsi Wakho study, a prospective cohort of ALHIV. Adolescents aged (10‐19) years at baseline were recruited from a municipality in Eastern Cape Province in South Africa, a province with an estimated overall HIV prevalence of 14% [23]. Participants were recruited by identifying all adolescents initiated on treatment in the area through medical records in all 52 ART‐providing public health facilities and were traced in their communities, homes, or schools. HIV‐negative peers from neighboring homes and some co‐resident adolescents were also interviewed to minimize stigma. Baseline interviews were conducted in 2014–2015, with follow‐up interviews in 2016–2017 and 2017–2018. Adolescents provided information on self‐reported adherence using questionnaires co‐developed, informed by qualitative work done as part of Mzantsi Wakho, and piloted among adolescents affected by HIV in the study area. Concurrently, during the first two waves of data collection, biomarker data from clinic records were extracted from 52 healthcare facilities in the province where ALHIV had received care [24].

Voluntary informed consent was obtained from adolescents and caregivers when adolescents were <18 years old. Ethical approvals for the Mzantsi Wakho study were granted by the University of Cape Town (UCT/CSSR/2013/4) and (UCT/CSSR/2019/01), Oxford University (CUREC2/12‐21), provincial Departments of Health and Education, and ethical review boards of participating healthcare facilities.

### 2.1 Measures

Our main variable *– past‐week adherence –* was defined based on indicators of currently taking ART and not having missed any doses in the past seven days (including weekdays and weekends) [25]. If the participant reported missing any dose or currently not taking ART (i.e. defaulting) we classified them as non‐adherent. We computed *sustained adherence* as 1 if the participant reported past‐week adherence at all the three waves of data collection and 0 otherwise (*inconsistent adherence)*. Viral load measurements, closest to the interview date, obtained from adolescents' clinic records were used to validate the self‐reported past‐week measure of adherence using study Wave 1 and Wave 2, for which viral loads were available. Due to limited testing, about 60% of adolescents with an available viral load had a measurement from the two years before or after the interview date [26]. Socioeconomic factors included adolescent *age, sex, urban/rural location, orphanhood*, and living in *formal or informal housing*‐ based on whether the adolescent reported living in an informal house‐shack. We also included a measure of *household poverty*, defined as lacking access to any of the eight basic necessities, namely: food, clothing, doctor, fees, shoes, toiletries, uniform, and school equipment selected by over 80% of the South African population in a nationally representative survey [27]. HIV‐related factors included *mode of HIV acquisition* (vertical/horizontal) and *adolescent health self‐rating*. Mode of HIV acquisition was defined using an age cut‐off, validated with a detailed algorithm in the absence of definitive clinic notes ascribing mode of HIV acquisition. Adolescents who began ARVs before the age of 10 were designated as vertical transmission and those who began ARVs at 10 years old and after were designated as horizontal transmission [28, 29].

### 2.2 Analyses

First, we validated our adherence measure by using a random‐effects logistic regression model to assess the association between non‐detectable viral load (<50 copies/mL) and past‐week adherence, controlling for other socio‐demographic factors. In this analysis, we used the random‐effects model to utilize the repeated measures structure of the data (data from the same subjects at two time‐points) as well as include time‐invariant factors like sex and mode of HIV acquisition. This step also included attrition analysis comparing baseline characteristics of complete versus missing viral load observations. Second, in our main analysis, we describe the prevalence of past‐week adherence at each wave and then computed the rates of cumulative sustained adherence across the three waves. We further compared baseline sociodemographic variables, including mode of HIV acquisition of adolescents who reported sustained vs. inconsistent ART adherence, and evaluated the differences using the Chi‐square test statistic. Step two and the attrition analysis included data from the three waves of data collection, while step one included data from the first two waves, for which viral loads were available. All analyses were done in Stata v.16.

## 3 RESULTS AND DISCUSSION

### 3.1 Study sample

Out of the 1 046 ALHIV interviewed at baseline, 933 participants were retained at all three waves of data collection and were the focus of this analysis. This study had a 90% uptake, 94% retention at Wave 2, 97% retention at Wave 3, and 3.4% mortality. There were no significant differences between participants retained and those lost‐to‐follow‐up except that those lost to follow‐up were likely to be older (see Table S2). At baseline, the mean (SD) age was 13.6 ± 2.9 (range 10–19 years). A comparison of those with viral load and those missing viral load records (see Table S1 in Appendix A) showed that older adolescents with horizontally acquired HIV at baseline were likely to be missing a viral load record. Of those with a viral load record at baseline (n = 624), two‐thirds (63.4%) of the sample had a viral load of less than 50 copies/mL. The majority of the adolescents were female (55.1%), 26.6% lived in rural settings, and 18.4% in informal settlements. 14.9% of the adolescent were double orphans. A fifth of this sample has likely horizontally acquired HIV [29].

### 3.2 Validation of adherence measure

The past‐week adherence measure was validated by comparison with viral load from the first two waves where data were available (Table 1). Controlling for socio‐demographic characteristics, we found that adolescents who reported past‐week adherence were significantly more likely to have a non‐detectable viral load (aOR 1.72, 95% CI 1.14‐2.59, p = 0.009).

**Table 1:** Random‐effects model of the relationship between self‐reported past‐week adherence and patient file‐based non‐detectable viral load (n = 756; 1197 observations)

	Non‐detectable viral load (<50 copies/mL)^¥^			
Factors	OR (95%CI)	*P*‐value	aOR (95%CI)	p‐value
Past‐week adherence	1.80 (1.20‐2.70)	0.004	1.72 (1.14‐2.59)	0.009
Age	0.88 (0.81‐0.95)	0.002	0.89 (0.81‐0.97)	0.012
Female	0.91 (0.57‐1.47)	0.709	1.06 (0.66‐1.71)	0.804
Rural residence	0.61 (0.37‐1.01)	0.053	0.65 (0.39‐1.08)	0.099
Double Orphan	1.11 (0.61‐2.01)	0.727	1.34 (0.72‐2.51)	0.354
Informal housing	0.76 (0.44‐1.32)	0.327	0.80 (0.46‐1.40)	0.439
Poverty	0.61 (0.39‐0.95)	0.028	0.65 (0.41‐1.02)	0.060
Horizontally transmission	0.51 (0.28‐0.94)	0.031	0.87 (0.44‐1.71)	0.678
Self‐rated poor health	0.31 (0.12‐0.75)	0.009	0.31 (0.12‐0.77)	0.012

*aOR: odds ratio (adjusted).

*Copies/mL: The number of HIV copies in a milliliter of blood

*95% CI: 95% confidence intervals

*These are routine viral loads which are collected following the steps below after ART initiation (1) ART initiation and fast track initiation counseling, (2) 6‐month from ART initiation, viral load collected, followed by a 12‐month viral load, and (3) If the patient is stable and adherent on ART then 12 monthly routine viral load monitoring as highlighted in the South African National Consolidated Guidelines in 2020.

^¥^ Viral load data were available for the first two study waves and this is a sub‐sample analysis for only those with a viral load record. We also assessed for differences between those with a viral load record and those without a viral load record as shown in the supplementary files.

### 3.3 Prevalence of past‐week adherence

**Figure 1:**
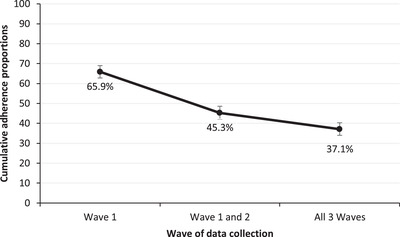
Past‐week cumulative sustained adherence rates among adolescents living with HIV retained across the three waves (N=933). **Notes**: Proportions were computed based on the same denominator (N=933) participants interviewed at all waves. 65.9% of participants who reported past‐week adherence at Wave 1, 45.3% of participants reported past‐week adherence at both Wave 1 and Wave 2 as a proportion on (N=933). Lastly, we computed the number of participants who reported past‐week adherence at all three waves (37.1%) as a fraction of (N=933) which is the proportion defined as sustained adherence.

The prevalence of adherence at baseline was 65.9%, 64.8% at Wave 2, and 75.0% at Wave 3 based on the past‐week adherence measure (see Figure S1 in Appendix). Whilst the past‐week adherence prevalence was stable at about 68% on average at each wave, the majority of adolescents reported inconsistent adherence over the three waves as shown in Figure 1. 65.9% reported past‐week adherence at baseline, and only 37.1% reported sustained past‐week adherence over the three waves.

### 3.4 Profile of adolescents with sustained adherence

Table 2 shows a comparison of baseline characteristics of sustained vs. inconsistent adherent adolescents. We found significant differences in sustained adherence by baseline age and the mode of HIV acquisition, but not the sex of the adolescents. Older age (p = 0.007) and horizontal transmission (p = 0.002) were associated with inconsistent adherence over the three waves.

**Table 2:** Descriptive statistics of sustained past‐week adherence by socio‐demographic characteristics, and mode of HIV acquisition among ALHIV (N = 933)

	Total (N = 933)	Inconsistent Adherence (N = 587)	Sustained Adherence (N = 346)	
Baseline factors	N (%)	N (%)	N (%)	p‐value
Age (15+ years)	337 (36.1)	231 (39.4)	106 (30.6)	0.007
Female	514 (55.1)	335 (57.1)	179 (51.7)	0.113
Horizontal transmission	197 (21.3)	142 (24.4)	55 (15.9)	0.002
Double Orphan	139 (14.9)	83 (14.1)	56 (16.2)	0.397
Informal housing	172 (18.5)	101 (17.2)	71 (20.6)	0.200
Poverty	633 (67.9)	397 (67.6)	236 (68.2)	0.856
Self‐rated poor health	52 (5.6)	38 (6.5)	14 (4.1)	0.118

^¥^ ALHIV‐ adolescents living with HIV

## 4 DISCUSSION

Overall, adherence rates in this study were relatively stable across time, with 65.9%, 64.8%, and 75.0% reporting past‐week adherence at Wave 1, 2, and 3, respectively. This study also showed that adolescence is marked with long‐term inconsistencies in adherence, for example, few adolescents (only 37.1%) of the sample (N = 933) reported sustained adherence across the three study waves which may owe to barriers to adherence described in other studies [15]. Our descriptive analysis also showed that inconsistent adherence was significantly associated with older age and horizontal HIV acquisition. This is consistent with prior research, which shows that older age is implicated since it is associated with increasing responsibility for self‐health care [13, 30]. Inconsistent adherence among adolescents with horizontally acquired HIV may be associated with failure to adapt to complicated ART medication routines and other related psychosocial problems, as highlighted in [13, 31, 32]. This difference in sustained adherence by mode of HIV acquisition may help inform health interventions aimed at improving the health of adolescents living with HIV.

Our findings also reflect that cross‐sectional assessments of adherence mask a significant lack of consistency in adherence in the long term. Furthermore, our study provides actual estimates of sustained adherence over three waves. It is also important to note that most participants’ clinic files in this sample were missing viral load measurements, which may reflect the challenges of point‐of‐care viral load monitoring in resource‐limited settings [33].

An important strength of the study is that the sample is based on a total sampling of the study area, which is helpful when estimating prevalence compared to hospital‐based studies. Self‐reported measures of adherence are prone to some level of bias related to social desirability and recall‐error, which may lead to overestimating adherence [34]. However, in this study, a measure of recent adherence (past week) was used to minimize recall bias. Although adherence varies significantly by measure, self‐reported measures remain the most widely used measures of adherence in both clinical and research settings [34]. In addition, for those adolescents whose clinic patient files contained a viral load in the relevant period, we found that self‐reported adherence significantly predicted viral suppression.

## 5 CONCLUSIONS

Sustaining ART adherence is a challenge for adolescents living with HIV as they get older, particularly those who recently acquired HIV, hence rigorous longitudinal assessments of adherence are required. Although much work has been done on adolescents with perinatally acquired HIV, more studies should focus on those with horizontally acquired HIV. Our findings highlight the urgent need for flexible adherence support to address the specific needs of adolescents, including barriers to ART adherence. There is also a need to ensure we do not lose older adolescents from care.

## FUNDING

The Mzantsi Wako Study was supported by the Evidence for HIV Prevention in Southern Africa (EHPSA); a UK Aid programme managed by Mott MacDonald, Janssen Pharmaceutica N.V., part of the Janssen Pharmaceutical Companies of Johnson & Johnson; the Nuffield Foundation (CPF/41513; OPD/31598), the Regional Inter‐Agency Task Team for Children Affected by AIDS‐Eastern and Southern Africa (RIATT‐ESA), the International AIDS Society through the CIPHER grant (155‐Hod; 2018/625‐TOS); and Claude Leon Foundation [F08 559/C]. Support for the authors and team is provided by the UKRI GCRF Accelerating Achievement for Africa's Adolescents Hub (Grant Ref: ES/S008101/1), the European Research Council (ERC) under the European Union's Horizon 2020 Research and Innovation Programme (grant agreement no. 771468), UNICEF Eastern and Southern Africa Office (UNICEF‐ESARO), Oak Foundation (R46194/AA001 & OFIL‐20‐057), the Fogarty International Center, National Institute on Mental Health, National Institutes of Health (Award Number K43TW011434). This study was also possible thanks to the UK Medical Research Council (MRC) and the UK Department for International Development (DFID) under the MRC/DFID Concordat agreement, and by the Department of Health Social Care (DHSC) through its National Institutes of Health Research (NIHR) [MR/R022372/1], University of Oxford's ESRC Impact Acceleration Account (IAA) [K1311‐KEA‐004 & 1602‐KEA‐189] and Clarendon‐Green Templeton College Scholarship, the Economic and Social Research Council [IAA‐MT13‐ 003], the John Fell Fund [103/757 & 161/033], the Leverhulme Trust [PLP‐2014‐095] and Research England.

## DISCLAIMER

The content is solely the responsibility of the authors and does not necessarily represent the official views of the agencies, institutions or organizations listed.

## AUTHORS’ CONTRIBUTIONS

ET and LC designed and implemented the overall study. LC conceptualized the analyses. SZ led the analyses and write‐up of the manuscript. LC, ET, YS provided edits and feedback on manuscript content and have approved the final draft.

## COMPETING INTEREST

The authors declare that they have no competing interests.

## Supporting information


**Table S1**: Comparison of baseline indicators for complete and missing viral load measurements
**Table S2**: Comparison of baseline characteristics for complete cases and LFTU^*^ (Full sample)
**Figure S1**: Wave‐specific past‐week adherence rates among adolescents living with HIV (N=933)

## Data Availability

The data that support the findings of this study are available for non‐profit use upon reasonable request following study data sharing protocols available here: http://www.mzantsiwakho.org.za/publications.
