# Supplementary material for: Uncovering ART adherence inconsistencies: An assessment of sustained adherence among adolescents in South Africa
Source: J Int AIDS Soc. 2021 Oct 28;24(10):e25832. doi: 10.1002/jia2.25832 (PMC8552454; doi:10.1002/jia2.25832)
Supplement: Supplementary file 1 — Table S1: Comparison of baseline indicators for complete and missing viral load measurements Table S2: Comparison of baseline characteristics for complete cases and LFTU* (Full sample) Figure S1: Wave‐specific past‐week adherence rates among adolescents living with HIV (N=933) [file JIA2-24-e25832-s001.docx]

**Appendix**

**Table S1: Comparison of baseline indicators for complete and missing viral load measurements**

|  | **Viral load record** | | |  |
| --- | --- | --- | --- | --- |
|  | **Total**  **(N=933)** | **Complete**  **(N=624)** | **Missing**  **(N=309)** |  |
| **Baseline factors** | N (%) | N (%) | N (%) | p-value |
| Age (15+ years) | 337 (36.1) | 195 (31.3) | 142 (46.0) | **<0.001** |
| Female | 514 (55.1) | 330 (52.9) | 184 (59.6) | 0.054 |
| Rural | 248 (26.6) | 165 (26.4) | 83 (26.9) | 0.469 |
| Informal housing | 172 (18.4) | 122 (19.6) | 50 (16.2) | 0.171 |
| Double orphan | 139 (14.9) | 94 (15.1) | 45 (14.6) | 0.840 |
| Poverty | 633 (67.9) | 414 (66.4) | 219 (70.9) | 0.163 |
| Health poor (self-rating) | 52 (5.6) | 34 (5.5) | 18 (5.8) | 0.813 |
| Horizontal infected | 197 (21.1) | 109 (17.5) | 88 (28.5) | **<0.001** |
| Past-week adherence | 615 (65.9) | 435 (69.7) | 180 (58.3) | **<0.001** |

# **Table S2: Comparison of baseline characteristics for complete cases and LFTU^¥^ (Full sample)**

|  | **Total**  **(N=1046)** | **Complete cases** **(N=933)** | **Loss-to-follow up** **(N=113)** |  |
| --- | --- | --- | --- | --- |
| **Baseline characteristics** | **N (%)** | **N (%)** | **N (%)** | **P-value** |
| Past-week adherence | 681 (65.1) | 615 (65.9) | 66 (58.4) | 0.114 |
| Age (15+ years) | 394 (37.7) | 337 (36.1) | 57 (50.4) | **0.003** |
| Female | 576 (55.1) | 514 (55.1) | 62 (54.9) | 0.964 |
| Rural residence | 271 (25.9) | 248 (26.6) | 23 (20.4) | 0.509 |
| Informal housing | 196 (18.7) | 172 (18.4) | 24 (21.2) | 0.728 |
| Orphanhood | 616 (58.9) | 545 (58.4) | 71 (62.8) | 0.367 |
| Sexually infected | 222 (21.2) | 197 (21.1) | 25 (22.1) | 0.689 |
| Poverty | 708 (67.7) | 633 (67.9) | 75 (66.4) | 0.752 |
| ^¥^ LFTU represents adolescents lost-to-follow-up at least one-time point  *At each successive wave, participants were actively followed up. Below is the breakdown of the (N=113) loss-to-follow up between Wave 1 and 3: (N=12) passed on between Wave 1 and 2, and (N=22) passed on between Wave 2 and 3, (N=55) were lost-to-follow-up for the following reasons between Wave 1 and Wave 2:- refusals, untraceable or avoidant. (N=24) were lost-to-follow-up between Wave 2 and Wave 3 either as refusals, untraceable or avoidant. | | | | |

**Figure S1: Wave-specific past-week adherence rates among adolescents living with HIV (N=933)**
